# Accuracy of diagnosis among clinical malaria patients: comparing microscopy, RDT and a highly sensitive quantitative PCR looking at the implications for submicroscopic infections

**DOI:** 10.1186/s12936-023-04506-5

**Published:** 2023-03-04

**Authors:** Stephen Opoku Afriyie, Thomas Kwame Addison, Yilekal Gebre, Abdul-Hakim Mutala, Kwasi Baako Antwi, Dawood Ackom Abbas, Kofi Agyapong Addo, Austine Tweneboah, Nana Kwame Ayisi-Boateng, Cristian Koepfli, Kingsley Badu

**Affiliations:** 1 Department of Theoretical and Applied Biology, Kwame Nkrumah University of Science and Technology (KNUST), Kumasi, Ghana; 2 Department of Biological Sciences, University of Notre Dame, South Bend, IN USA; 3 Infectious Disease Unit, University Hospital, KNUST, Kumasi, Ghana

**Keywords:** Malaria, Microscopy, Rapid diagnostic test, varATS qPCR, Sensitivity

## Abstract

**Background:**

The World Health Organization recommends parasitological confirmation of all suspected malaria cases by microscopy or rapid diagnostic tests (RDTs) before treatment. These conventional tools are widely used for point-of-care diagnosis in spite of their poor sensitivity at low parasite density. Previous studies in Ghana have compared microscopy and RDT using standard 18S rRNA PCR as reference with varying outcomes. However, how these conventional tools compare with ultrasensitive *var*ATS qPCR has not been studied. This study, therefore, sought to investigate the clinical performance of microscopy and RDT assuming highly sensitive *var*ATS qPCR as gold standard.

**Methods:**

1040 suspected malaria patients were recruited from two primary health care centers in the Ashanti Region of Ghana and tested for malaria by microscopy, RDT, and *var*ATS qPCR. The sensitivity, specificity, and predictive values were assessed using *var*ATS qPCR as gold standard.

**Results:**

Parasite prevalence was 17.5%, 24.5%, and 42.1% by microscopy, RDT, and *var*ATS qPCR respectively. Using *var*ATS qPCR as the standard, RDT was more sensitive (55.7% vs 39.3%), equally specific (98.2% vs 98.3%), and reported higher positive (95.7% vs 94.5%) and negative predictive values (75.3% vs 69.0%) than microscopy. Consequently, RDT recorded better diagnostic agreement (kappa = 0.571) with *var*ATS qPCR than microscopy (kappa = 0.409) for clinical detection of malaria.

**Conclusions:**

RDT outperformed microscopy for the diagnosis of *Plasmodium falciparum* malaria in the study. However, both tests missed over 40% of infections that were detected by *var*ATS qPCR. Novel tools are needed to ensure prompt diagnosis of all clinical malaria cases.

**Supplementary Information:**

The online version contains supplementary material available at 10.1186/s12936-023-04506-5.

## Background

Malaria remains a major public health concern, particularly in sub–Saharan Africa where > 93% of global malaria cases and deaths occur annually [1]. Malaria is endemic across all sixteen administrative regions in Ghana with the entire population at risk. Transmission is year-round and heterogeneous among different ecological zones within the country—ranging from hyperendemicity in the Upper West Region, hypo-endemicity in Greater Accra, and meso-endemicity in the forest and southern coastal areas [2, 3]. Ghana has seen dramatic progress in its fight against malaria, evidenced by over 50% and 65% decline in morbidity and mortality respectively between 2005 and 2015 [4]. Despite the efforts made to overcome malaria in Ghana, the disease remains the leading cause of hospitalizations, accounting for over 30% and 23% of all outpatient and inpatient admissions, respectively, across the country [4].

In line with World Health Organization (WHO) policy of test before treatment, the National Malaria Control Programme in Ghana promotes the usage of microscopy or rapid diagnostic tests (RDTs) for routine malaria diagnosis [2]. Microscopy is generally inexpensive to operate and able to distinguish between the various *Plasmodium* species [5]. Additionally, microscopy is useful to estimate parasite density, monitor drug efficacy and is capable of diagnosing other infections. However, the accuracy of microscopy is variable and largely dependent on the expertise of the microscopist and the quality of staining reagents [6]. The limit of detection of microscopy is generally estimated as 50–500 parasites/µL of blood [7] though the expert microscopist may detect 10 parasites/μl of blood [8]. Microscopy is also poorly suited for population-level surveillance due to its labor intensive process [9].

The advent of rapid diagnostic tests and its adoption in Ghana has led to a significant reduction in presumptive diagnosis across the country [10]. RDTs provide quicker results (~ 15-20 min) than microscopy, are easy to perform, and do not require electricity, thus offering a good alternative in resource-limited settings [11]. Nevertheless, RDTs are only qualitative and reportedly yield more false positives (than microscopy) due to persistent circulation of antigens (HRP-2) even after parasite clearance by anti-malarials [12]. Studies have shown most RDTs to be less sensitive at low parasite densities (< 200 parasites/μL), thus missing chronic latent infections in asymptomatic populations, particularly in low-transmission settings [13, 14]. The accuracy of HRP-2 based RDT may also be limited by mutant or *hrp2* gene deletions, thus leading to false negatives [15, 16]. The WHO recommends policy change away from HRP-2 based RDTs when false negative test results due to *hrp2/hrp3* gene deletions in clinical malaria cases exceeds 5% [17].

Nucleic acid amplification tests (NAATs) provide by far the highest sensitivity and specificity for malaria diagnosis. Commonly used NAATs include Polymerase Chain Reaction (PCR) and Loop-mediated Isothermal Amplification (LAMP) that detect parasite DNA in blood [18]. The limit of detection of NAATs range between < 1–5 parasites/μl, hence their ability to detect low-density infections [19, 20]. However, NAATs are poorly suited for routine diagnosis due to increased costs and are best used in epidemiological surveys and also as reference standards in diagnostic accuracy studies [9].

Several diagnostic accuracy studies in Africa and Asia have compared microscopy and RDT using conventional or nested 18S rRNA PCR as reference [15, 19, 21–24]. Recently, a novel qPCR assay that targets the *var* gene family of *P. falciparum* has been developed and proven to be 10 times more sensitive than conventional 18S rRNA PCR [25]. The multi copy *var* gene family is mostly located in sub telomeric regions where it encodes *P. falciparum* erythrocyte membrane protein 1 (*Pf*EMP1) [26]. About 59 different *var* genes have been identified in the genome of 3D7 culture strain. *var* genes possess intracellular acidic terminal sequences (ATS) with domains which are well-conserved. The limit of detection of *var*ATS qPCR has been reported as 0.03 parasites/μl of blood [25, 27].

Whiles most studies in Ghana have compared microscopy and RDTs using conventional PCR methods as reference [10, 28, 29], there is currently no information on the performance of these traditional malaria diagnostic tools against the highly sensitive *var*ATS qPCR assay. This study provides baseline information on how the commonly used malaria tests in Ghana perform against *var*ATS qPCR. As Ghana set ambitious targets from malaria control to elimination [30], active monitoring of the accuracy of routinely used diagnostic tests is necessary to track progress and guide national policy. It is also important that low density submicroscopic malaria infections that are undetected are identified and treated to interrupt disease transmission. In this study, the clinical performance of microscopy and RDT was assessed using *var*ATS qPCR as gold standard. The study also aimed to shed light on the burden of missed and submicroscopic infections among clinical malaria patients in Ghana.

## Methods

### Study areas

**Fig. 1:**
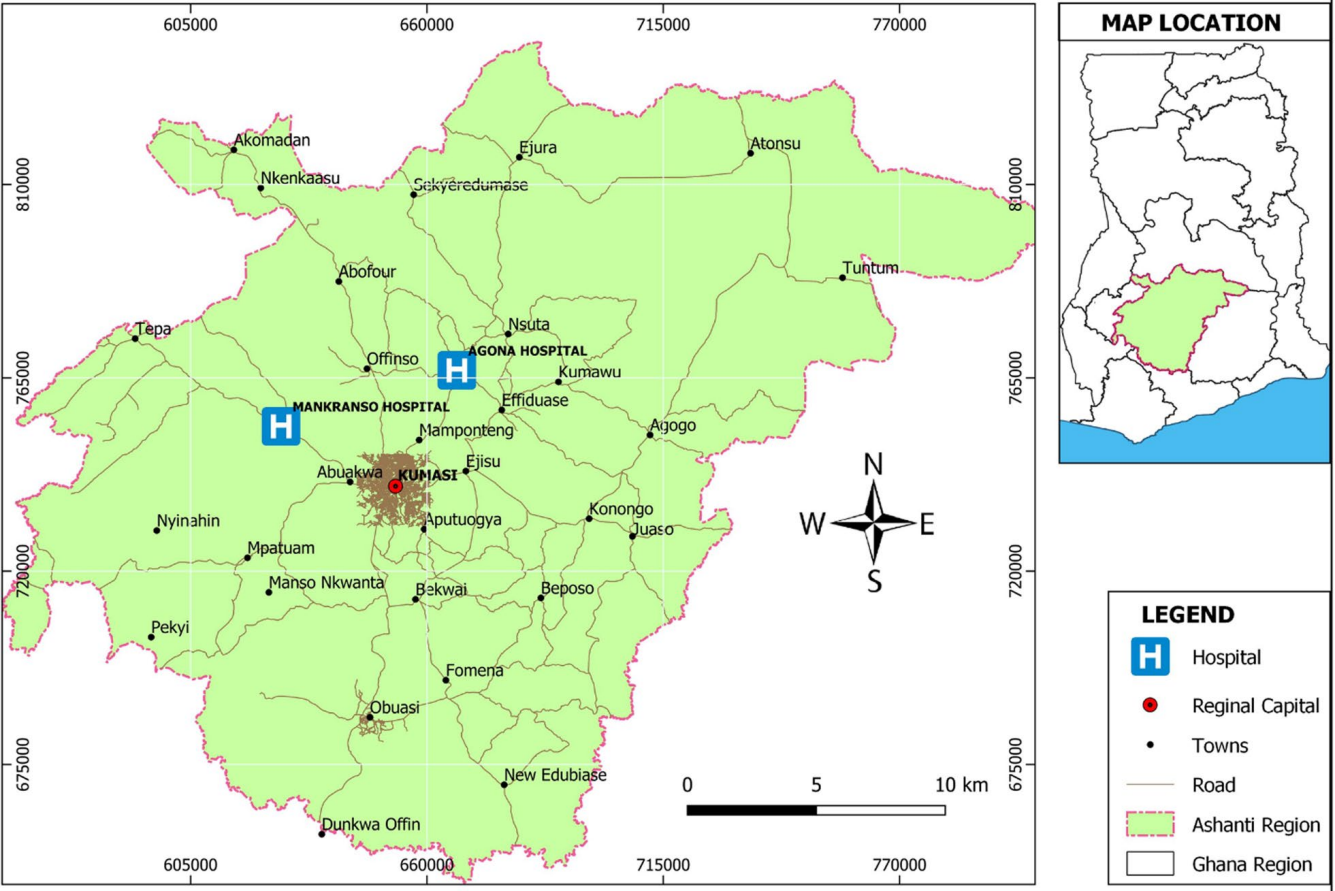
A map showing the location of the study areas in the Ashanti Region of Ghana. [The map was created by Mr. Ema Dari of the Department of Geography and Rural Development, KNUST using ArcGIS Desktop 10.6.1 software]

The study was conducted at two primary health centers in the Ashanti Region of Ghana (Fig. 1), namely Agona Government Hospital (AGH) and Mankranso Government Hospital (MGH). AGH is situated at Agona, the administrative capital of the Sekyere South District in Ghana, where it serves as the main referral health facility for surrounding villages and towns (latitude 6^o^50’N and longitude 1^o^29’W). About 47% of the inhabitants live in rural areas and 67% are involved in agriculture. The mean annual rainfall ranges between 855 mm and 1500 mm with a daily warm to hot temperature at about 27 °C [31].

Mankranso Government Hospital is located at Mankranso, the capital of the Ahafo Ano South West District (formerly Ahafo Ano South District) in the Ashanti Region of Ghana. According to the 2010 Population and Housing Census, about 90% of the total population live in rural areas. Approximately 81.7% percent of indigenous households in Mankranso are engaged in crop farming whiles others are actively involved in poultry farming. The district is located at latitude 6^o^42’N and longitude1^o^45’W. Mankranso has a wet semi-equatorial climate with a mean monthly temperature between 26 °C–28 °C with two major rainfall patterns in the district [32].

### Study design

This was a cross-sectional hospital-based study conducted between January and June 2021. Sampling was done three days per week between the hours of 9 a.m. and 3 p.m. each day. Inclusion criteria for the study were suspected malaria patients (i.e., those referred to the laboratory for malaria diagnosis by clinicians) of all ages and gender. Written informed consent was obtained from all participants who were ≥ 18 years old. Parental or guardian consent was obtained for all those who were below 18 years old. Unsuspected malaria patients and participants who could not provide informed consent were excluded from the study.

### Sample size

The sample size was pre-calculated as the minimum number of samples required to attain 95% sensitivity and specificity assuming a 5% margin of error at 95% confidence level (α = 0.05, power 1 – *β* = 0.20*)* as previously described [33–35]. To calculate the final sample size, a previous prevalence of 25.96% was used [36]. After substituting in the variables, a minimum sample size of 379 was required for the study.

### Study procedures

A well-structured questionnaire was administered to participants in layman’s terms to capture socio-demographic and clinical information (including fever history). Afterwards, 2 ml of venous blood was collected from participants into EDTA tubes. RDT was immediately performed for all participants and blood smears (thick and thin) were prepared for microscopic diagnosis. 1 ml aliquots were also taken for diagnosis by qPCR and transported in cold boxes to the Vector-Borne Infectious Diseases laboratory at Kwame Nkrumah University of Science and Technology (KNUST), Kumasi, Ghana, for storage and further analysis.

### Laboratory investigations

#### Rapid diagnostic testing

The CareStart™ Malaria Pf (HRP2) Ag RDT (AccessBio, USA) kit was chosen for the present study since it passed WHO-recommended criteria for routine diagnosis [37]. Briefly, the RDT was labelled with a unique participant code and date before 5µL of whole blood was pipetted into the sample well, “S”, on the RDT cassette. Afterwards, two drops (60µL) of buffer solution provided by the manufacturer were added to the well labelled “A” on the cassette. Test results were recorded after twenty (20) minutes according to the manufacturer’s instructions.

#### Diagnosis by microscopy

Duplicate thick and thin blood films were prepared for each participant on clean, well-labelled, frosted glass slides. Briefly, 2µL and 6µL of whole blood were pipetted to prepare thin and thick films respectively on the slide as previously described [38]. The thin smears were fixed in absolute methanol after which the slides were arranged in slide boxes for further analysis. All slides were stained using 10% Giemsa working solution and subsequently imaged at the × 100 objective. Parasite detection was done by examining at least 100 high power fields. Parasite quantification was estimated based on the total number of malaria parasites counted in 200 or 500 white blood cells and then multiplied by 8000 white blood cells (WBCs) as previously described [39]. Microscopy diagnosis was done by two independent WHO-certified (Level 1) experts who were blinded to both RDT and each other’s result. Slides were adjudged “positive” if positive by either or both of the microscopists. Final parasite density estimates were obtained by taking the average of parasitaemia by both microscopists.

#### Molecular diagnosis by *var*ATS qPCR

DNA was extracted from 200 µL whole blood using the Macherey–Nagel NucleoMag extraction kit and eluted in 100 µL buffer. qPCR was done on a ThermoFisher QuantStudio 3 instrument in a total volume of 12 µL, including 4µL eluted DNA, corresponding to 8 µL blood using the highly sensitive *P. falciparum var* acidic terminal sequence (*var*ATS) multi-copy gene assay. The reaction mixture was made of 1.28μL of PCR water, 6μL of 1 × Perfect tough PCR MasterMix, 0.48μL of 10uM forward and reverse primers, 0.24μL of 10uM probe, and 4μL eluted DNA. The forward, reverse, and probe sequence used have been previously described [25]. Cycling conditions of the qPCR consisted of pre-incubation at 50 °C for 2 min, initial denaturation at 95 °C for 2 min, final denaturation at 95 °C for 10 s, annealing, and elongation at 55 ^℃^ for 30 s. Amplification of the target gene was done at 45 cycles. Parasite density was estimated using a standard curve of purified parasite genomic DNA (gDNA) quantified by droplet digital PCR of a tenfold dilution of 3D7 parasite culture (10^3^, 10^2^, 10^1^, 10^0^, and 10^–1^ copies/μl in double-distilled water). The limit of detection of qPCR was < 0.1 parasites/µL blood.

### Data analysis

Data from the questionnaire and laboratory results were coded and entered using Microsoft Excel 2016. The data was analyzed using GraphPad Prism 9.0 (San Diego, California) at 95% confidence level and a significance at p < 0.05. 2 × 2 contingency tables were drawn after which sensitivity, specificity, positive predictive value, and negative predictive values were calculated. Descriptive statistics were performed for socio-demographic features which were represented as frequencies and percentages. Mean or median was used to summarize quantitative data. Parasite density across the age groups was computed using ANOVA (geometric mean) and post-hoc analysis using Tukey’s multiple comparison test. The agreement between different diagnostic tests was calculated using the Kappa (κ) measure of inter-rater agreement. Briefly, κ < 0.20 indicated poor agreement, 0.21–0.40 fair, 0.41–0.60 moderate, 0.61–0.80 good, 0.81–0.99 very good and 1.00 indicate perfect agreement [40].

## Results

### Socio-demographic characteristics of the study population

Of the 1,040 participants recruited for the study, 458 (44.0%) were obtained from Mankranso Government Hospital (MGH) with the remaining 582 (56.0%) from Agona Government Hospital (AGH). All data is presented for both sites combined. Participants' age ranged between 3 weeks and 96 years with a median age of 22 years (IQR = 7 – 36 years). Majority of the study populace fell within the 15–30 age group (34.9%), followed closely by the > 30 (32.4%), < 5 (20.3%), and 5–14 (12.4%) age groups in descending order. Females were more than twice (70.7%) the number of males involved in the study (29.3%). Studentship/apprenticeship was the predominant occupation (33.0%) of the participants whereas civil servants (6.1%) were the least represented in the study (Table 1).

**Table 1:** Socio-demographic features of the study population

Baseline characteristics	Study area		Both sites, n (%)
	MGH, n (%)	AGH, n (%)	
Gender			
Male	114 (24.9)	191 (32.8)	305 (29.3)
Female	344 (75.1)	391 (67.2)	735 (70.7)
Age group (years)			
< 5	74 (16.2)	137 (23.5)	211 (20.3)
5–14	28 (6.1)	101 (17.4)	129 (12.4)
15–30	209 (45.6)	154 (26.5)	363 (34.9)
> 30	147 (32.1)	190 (32.6)	337 (32.4)
Occupation			
Farming	57 (12.6)	64 (11.0)	121 (11.7)
Trading	52 (11.5)	85 (14.6)	137 (13.2)
Civil servant	46 (10.2)	17 (2.9)	63 (6.1)
Student	114 (25.2)	227 (39.0)	341 (33.0)
Unemployed	83 (18.4)	44 (7.6)	127 (12.3)
Other	100 (22.1)	145 (24.9)	245 (23.7)
ITN usage			
Yes	275 (61.0)	215 (37.0)	490 (47.5)
No	176 (39.0)	366 (63.0)	542 (52.5)
History of fever			
Yes	307 (67.0)	386 (66.3)	693 (66.6)
No	151 (33.0)	196 (33.7)	347 (33.4)

### Parasite prevalence by microscopy, RDT, and *var*ATS qPCR

**Fig. 2:**
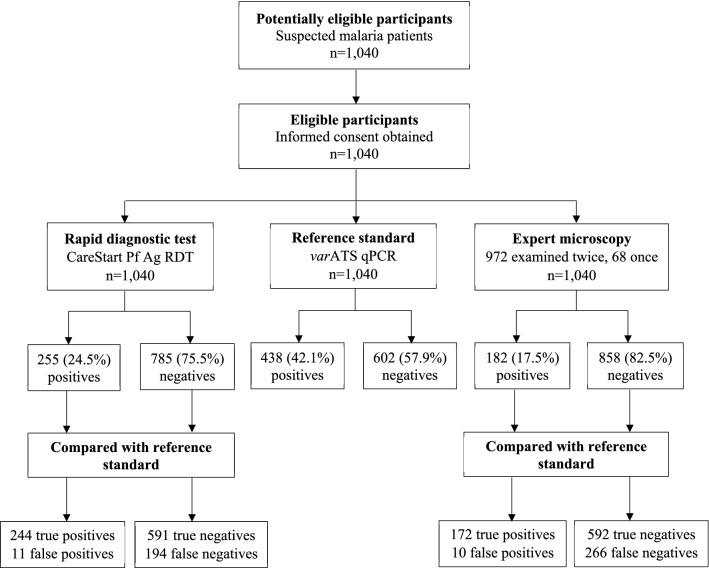
Flow chart describing participant recruitment and diagnostic tests performed

All 1,040 samples were tested for malaria by RDT, microscopy, and qPCR (Fig. 2). All but 68 slides were examined by two experts with an agreement of 96% (kappa value = 0.854, 95% CI 0.810–0.899) (Additional file 1). Unsurprisingly, higher parasite prevalence was detected by qPCR (42.1%) than RDT (24.5%) and microscopy (17.5%). By qPCR, higher parasite prevalence was recorded at MGH than AGH (Table 2).

**Table 2:** Parasite prevalence by RDT, microscopy, and *var*ATS qPCR

Test	Study area		Total (n = 1040)
	MGH (n = 458)	AGH (n = 582)	
	MP + (%)	MP + (%)	MP + (%)
RDT	104 (22.7)	151 (25.9)	255 (24.5)
Microscopy	66 (14.4)	116 (19.9)	182 (17.5)
qPCR	195 (42.6)	243 (41.8)	438 (42.1)

MP +  = Malaria positive

### Comparing diagnostic accuracy of RDT and Microscopy using qPCR as reference

**Fig. 3:**
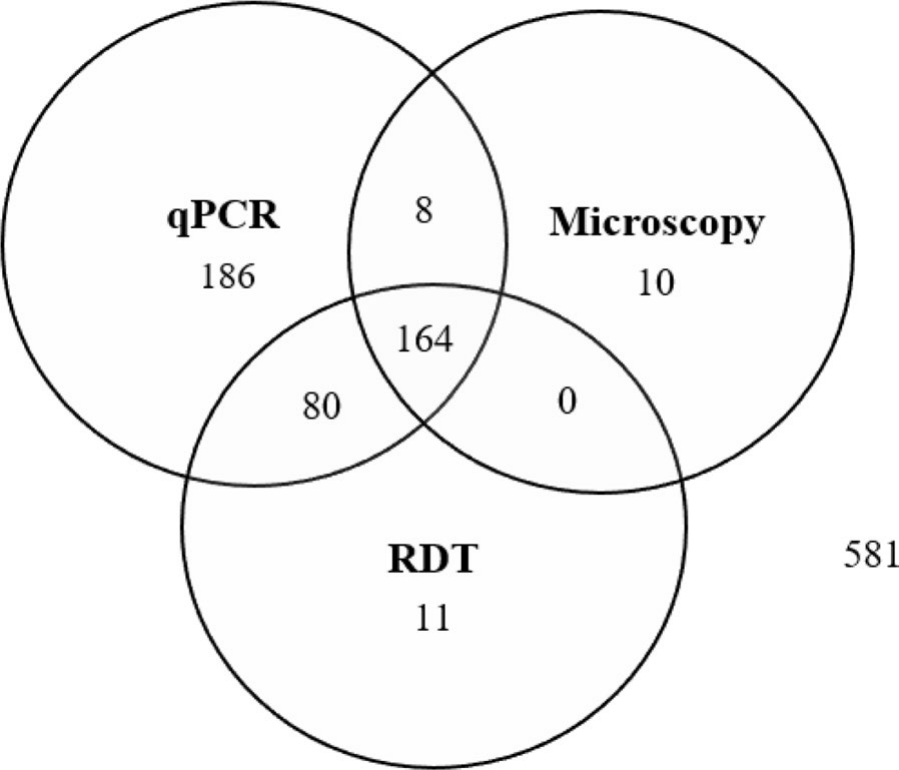
Detection of *P. falciparum* by microscopy, RDT, and *var*ATS  qPCR

Using highly sensitive *var*ATS qPCR as the reference standard, 438/1,040 patients were positive for *P. falciparum*. Out of the 438 patients positive by qPCR, RDT identified 244 (55.7%) infections whereas microscopy could detect only 172 (39.3%) true positives. Similar false positive rates were recorded in RDT, 11 (1.8%) and microscopy, 10 (1.7%) (Fig. 3). Microscopy reported a higher false negative rate (60.7%), missing out on 266 qPCR-positive samples than RDT which had a false negative rate of 44.3%, failing to identify 194 qPCR-positive samples.

The sensitivity of microscopy and RDT was 39.3% and 55.7% respectively. Both tests had comparable specificity (98.2% vs 98.3%), whiles RDT reported similar PPV (95.7% vs 94.5%) but higher NPV (75.3% vs 69.0%) than microscopy. Ultimately, RDT showed “moderate” agreement with qPCR (κ = 0.571), whereas microscopy showed “fair” (κ = 0.409) agreement (Table 3). The better sensitivity of RDT was particularly pronounced at low parasite density (qPCR < 200mps/μl) where RDT was found to be fourfold more sensitive than microscopy (27.9% vs 6.6%) and once again showed better agreement (κ = 0.326) with qPCR than microscopy (κ = 0.067) (Table 4).

**Table 3:** Diagnostic accuracy of microscopy and RDT using qPCR as reference

Performance metric	Test	
	RDT	Microscopy
TP (qPCR = 438)	244	172
FP (qPCR negative)	11	10
TN (qPCR = 602)	591	592
FN (qPCR positive)	194	266
Sensitivity % (95% C.I.)	55.7 (51.0–60.3)	39.3 (34.8–43.9)
Specificity % (95% C.I.)	98.2 (96.8–99.0)	98.3 (97.0–99.1)
PPV % (95% C.I.)	95.7 (92.4–97.6)	94.5 (90.2–97.0)
NPV % (95% C.I.)	75.3 (72.2–78.2)	69.0 (65.8–72.0)
Accuracy %	80.3	73.5
kappa value (95% C.I.)	0.571 (0.523–0.620)	0.409 (0.359–0.458)

*TP* True Positive, *FP* False Positive, *FN* False Negative, *TN* True Negative, *PPV* Positive Predictive Value, *NPV* Negative Predictive Value

**Table 4:** Diagnostic accuracy of microscopy and RDT at low parasite density (qPCR < 200p/µL)

Performance metric	Test	
	RDT	Microscopy
TP (qPCR = 244)	68	16
FP (qPCR negative)	11	10
TN (qPCR = 602)	591	592
FN (qPCR positive)	176	228
Sensitivity % (95% C.I.)	27.9 (22.6–33.8)	6.6 (4.1–10.4)
Specificity % (95% C.I.)	98.2 (96.7–99.0)	98.3 (97.0–99.1)
PPV % (95% C.I.)	86.1 (76.8–92.0)	61.5 (42.5–77.6)
NPV % (95% C.I.)	77.1 (74.0–79.9)	72.2 (69.0–75.2)
Accuracy %	77.9	71.9
kappa value (95% C.I.)	0.326 (0.260–0.392)	0.067 (0.023–0.111)

*TP* True Positive, *FP* False Positive, *FN* = False Negative, *TN* True Negative, *PPV* Positive Predictive Value, *NPV* Negative Predictive Value

### Accuracy of RDT and microscopy across different age groups using qPCR as reference

Across the different age groups, similar sensitivity and specificity trends were observed by both RDT and microscopy. In both tests, sensitivity and specificity were highest in the 5–14 age group followed by the < 5, 15–30, and > 30 age group in decreasing order. Both tests recorded > 94% specificity across the different age groups (Table 5).

**Table 5:** Diagnostic accuracy of microscopy and RDT across the age groups

Age group (years)	Sensitivity %	Specificity %	PPV %	NPV %
RDT				
< 5	67.0 (57.2–75.6)	99.1 (95.2–100.0)	98.5 (91.9–100.0)	77.9 (70.5–83.4)
5–14	87.8 (78.5–93.5)	94.6 (85.2–98.5)	95.6 (87.8–98.8)	85.3 (74.3–92.0)
15–30	48.7 (40.9–56.5)	98.1 (95.2–99.3)	94.9 (87.7–98.0)	72.2 (66.7–77.1)
> 30	34.5 (26.4–43.7)	98.7 (96.1–99.6)	92.9 (81.0–97.5)	74.9 (69.7–79.5)
Microscopy				
< 5	44.3 (34.9–54.2)	99.1 (95.2–100.0)	97.7 (88.2–100.0)	67.7 (60.2–74.3)
5–14	60.8 (49.4–71.1)	98.2 (90.4–100.0)	97.8 (88.7–99.9)	65.1 (54.3–74.4)
15–30	37.0 (29.8–44.9)	99.0 (96.6–99.8)	96.6 (88.5–99.4)	68.1 (62.7–73.1)
> 30	23.9 (17.0–32.5)	97.3 (94.3–98.8)	81.8 (65.6–91.4)	71.7 (66.4–76.5)

Values in the bracket indicate the 95% confidence interval

### Sensitivity of microscopy and RDT across different parasite densities by *var*ATS qPCR

As expected, higher sensitivity values were recorded with increasing parasite density. Parasitaemia level was defined as very low (< 100 parasites/µl), low (100–999 parasites/µl), moderate (1000–4999 parasites/µl), high (5000–99,999 parasites/µl), and hyper (≥ 100,000 parasites/µl blood) as previously described [41]. Interestingly, RDT showed superior sensitivity over microscopy at all parasitaemia levels (Table 6).

**Table 6:** Sensitivity across different parasite densities

Parasite density (parasites/µl of blood)	No. of cases	Sensitivity % (n)	
		Microscopy	RDT
Very low (< 100)	230	6.1 (14)	26.1 (60)
Low (100–999)	52	36.5 (19)	61.5 (32)
Moderate (1000–4999)	35	65.7 (23)	88.6 (31)
High (5000–99,999)	86	95.3 (82)	100.0 (86)
Hyperparasitaemia (≥ 100,000)	35	97.1 (34)	100.0 (35)

### Parasite prevalence across the age groups

**Fig. 4:**
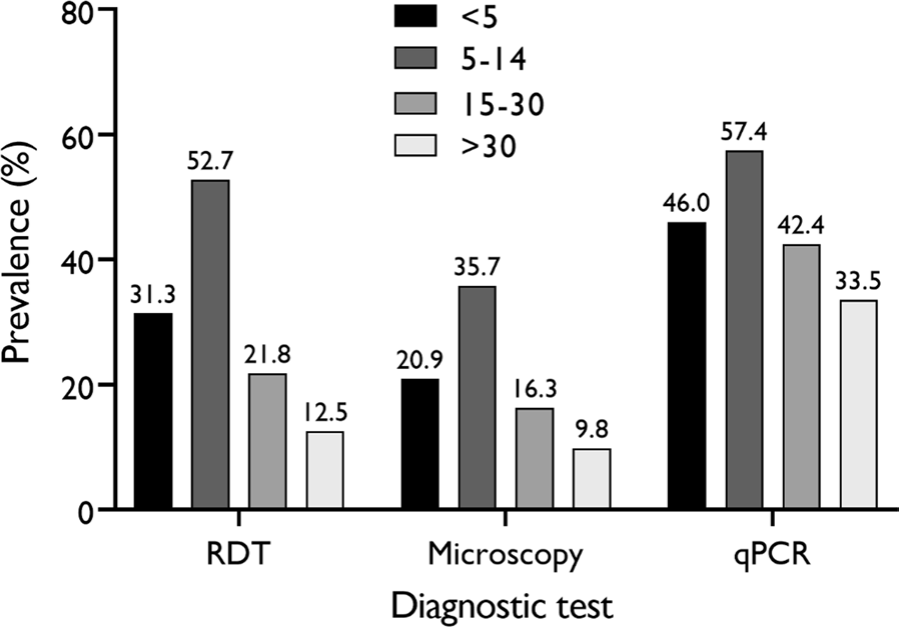
Parasite prevalence across different age groups

All three different tests detected high positivity rate among the 5–14 age group, with values that were distinctly higher than the other age groups (Fig. 4). The lowest prevalence was recorded in the > 30 age group by all three tests.

### Parasite density by *var*ATS qPCR across the age groups

**Fig. 5:**
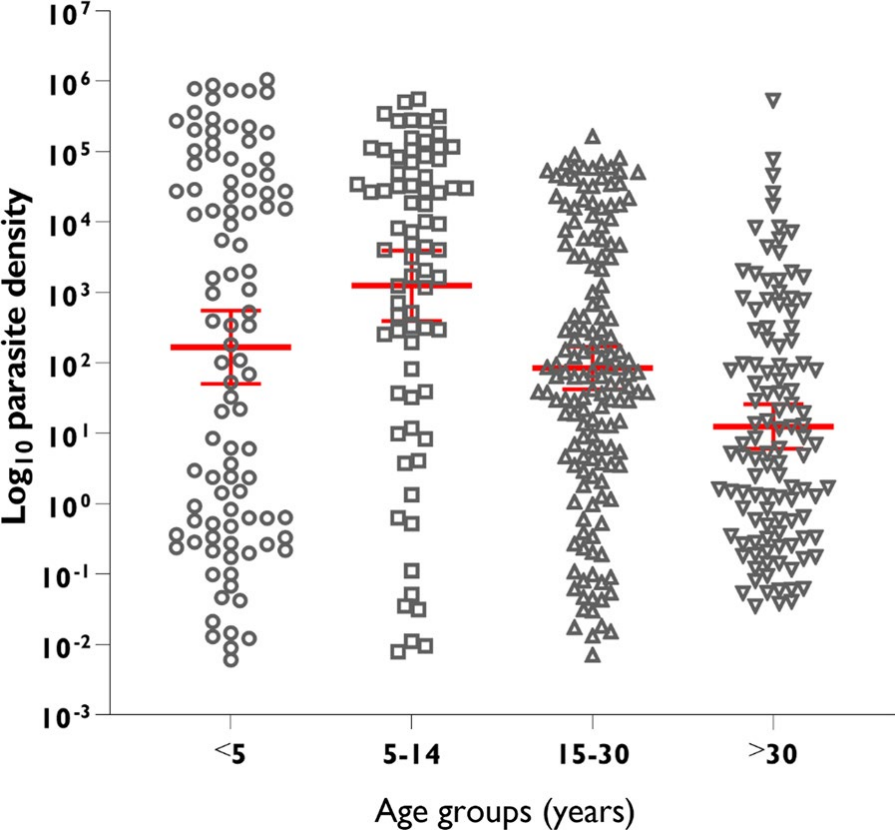
Parasite density across different age groups (error bars showing geometric mean with 95% CI)

A significant difference (p < 0.0001) in parasite density estimation was observed across the age groups. The geometric mean parasite density in the 5–14 age group (1246p/μl) was sevenfold higher than in children < 5 years (166.8p/μl), and 100-fold higher than that of adults > 30 years (12.4p/μl). The 15–30 age group had a geometric mean parasite density of 84.3p/μl. Figure 5 illustrates log_10_ transformation of parasite density by qPCR across the age groups.

## Discussion

Prompt and accurate diagnosis of malaria is the fundamental first step to effectively identify, treat and interrupt disease transmission [42]. An excellent modelling analysis predicts that > 100,000 malaria-associated deaths would be averted by a 95% sensitive and 95% specific diagnostic tool that require minimal infrastructure. According to the model, > 300,000 malaria-related deaths and ~ 450 million unnecessary treatments may be prevented by a 90% sensitive and 90% specific diagnostic test that require no infrastructure [43].

In Ghana, like most African countries, microscopy and RDTs are commonly used for routine malaria diagnosis in healthcare centers. Whilst extensive studies have compared microscopy and RDT based on standard 18S rRNA PCR results, this study is the first in Ghana to make comparisons using *var*ATS qPCR as reference in a clinical setting. Most *P. falciparum* strains possess relatively fewer 18S ribosomal subunits (5–8 copies/genome) [44] than the multi-copy *var* gene family which has approximately 59–60 copies/genome [45, 46]. *var*ATS qPCR therefore has a very low limit of detection (~ 0.03–0.15 parasites/µL) and has been proven to be tenfold more sensitive than traditional 18S rRNA PCR methods [25]. It is important that such sensitive tools are used to routinely quantify submicroscopic infections and measure how well routine diagnostics are performing. This is particularly relevant even as Ghana sets up an ambitious parliamentary caucus to accelerate malaria elimination [30].

Comparisons between microscopy, RDT, and PCR need to consider vastly different limits of detection of qPCR. Extraction from whole blood results in better sensitivity compared to dried blood spots [47]. The corresponding volume of blood screened by qPCR ranges from < 0.1 µL [48] to ≥ 200 µL [49] when DNA is concentrated during extraction. Multi-copy genes such as *var*ATS offer superior sensitivity compared to single copy genes [50]. As a result, the limit of detection differs across several orders of magnitude. A more sensitive PCR will result in more low-density infections being detected, and thus a lower sensitivity of microscopy or RDT compared to PCR. For the current study, DNA was extracted from whole blood and a multi-copy target was amplified, resulting in a very low limit of detection.

The present study revealed that microscopy missed almost two-thirds (60.7%) of infections, whereas RDT missed nearly half (44.3%) of qPCR-positive (438) cases. This corroborates a previous study where microscopy missed 169 (57%) out of 295 *var*ATS qPCR-positive infections [25]. A similar study in Nigeria which compared microscopy and CareStart RDT used in this study revealed microscopy (30%) missed a higher proportion of 112 *var*ATS qPCR-positive infections than RDT (12%) [27].

False negatives are of huge concern since failure to treat diseased persons may lead to continued disease transmission, increased morbidity and mortality [15]. Though false negative HRP-2 based RDT test results may arise from* hrp2* gene deletions, this does not seem to be crucial in Ghana currently [16, 51]. It is evident that submicroscopic infections largely accounted for the high false negatives recorded by microscopy and RDT in the current study (Table 6). Submicroscopic infections tend to persist for several months without showing any typical symptoms and would need to be actively detected and targeted for treatment. This is of particular significance in low-transmission areas aiming for successful malaria elimination since persistent submicroscopic infections may sustain disease transmission [52, 53]. In high transmission areas in sub-Saharan Africa, there are conflicting reports on the clinical relevance of submicroscopic infections. A cross-sectional survey in Malawi found no association between submicroscopic infection and clinical manifestation of malaria [54] whereas a recent study in Bagamoyo, Tanzania, was inconclusive on the contribution of submicroscopic infections to malaria transmission [55]. Meanwhile, longitudinal studies in Uganda found significant association between submicroscopic infections and febrile/non-febrile illnesses in younger children but not in adults [56]. Also, reports from South and Central Sudan associated maternal anemia and low birth weight with submicroscopic infection in pregnancy [57, 58]. Submicroscopic infections have been reported in multiple studies in Ghana [59, 60] however, its contribution to disease transmission and clinical manifestation is poorly understood and warrants further studies. New evidence suggests that submicroscopic infections disproportionally contribute to higher investment in gametocyte formation that may be picked up by vectors to sustain disease transmission [61]. This makes it even more important to understand the burden of subpatent infections in symptomatic people attending hospital to receive care.

Contrary to the high false negative rates observed in the study, both microscopy (10) and RDT (11) reported fewer false positives. The false positive HRP2-based RDT results may be due to persistent circulation of HRP2 antigens (up to two weeks) after parasite clearance [9, 62, 63]. Other potential causes of false-positive RDT results include persistence of clinically irrelevant gametocytes and cross-reactions with other non-*P. falciparum* species [63]. Conversely, the false positive microscopy results observed may be due to misidentification of artifacts as parasites [64] and morphologically similar blood-stage non-*P*. *falciparum* species. In the present study, there was a single case of *P. ovale* microscopy-positive infection that was missed by *var*ATS qPCR and the HRP-2 based RDT, both of which are specific for *P. falciparum* infections.

From the study, RDT was more sensitive, equally specific, and was a better predictor of malaria than microscopy (Tables 3 and 4). Consequently, RDT showed overall higher accuracy and better agreement (κ = 0.571) with *var*ATS qPCR than microscopy (κ = 0.409). This finding corroborates a community-based study in Nigeria [27] where RDT was more sensitive (73.9% vs 63.0%) and strongly agreed (κ = 0.74 vs κ = 0.67) with *var*ATS qPCR than microscopy. The study in Nigeria also reported microscopy to be more specific than RDT (99.5% vs 97%), albeit a marginal difference was observed in the present study [27]. The relatively higher sensitivity of RDT could be attributed to the fact that RDT targets antigens (HRP2/HRP3) expressed by parasites and not actual parasites as in the case of microscopy, hence may be able to pick up low density infections which may have been missed by microscopy [62].

Analysis of diagnostic performance across age groups revealed that sensitivity and specificity were influenced by parasite density (Figs. 4 and 5). High sensitivity was recorded in the 5–14 age group who presented with the highest parasite prevalence and parasite density (Table 5). Conversely, sensitivity decreased with decreasing parasite density (Table 6) and increasing age group (Table 5). The decrease in sensitivity with increasing age group has been reported in previous studies [15, 65]. A plausible interpretation for this occurrence is the development of acquired immunity in older individuals (15–30 and > 30 age groups) due to frequent exposure, hence giving them the ability to control parasite multiplication to submicroscopic levels that may be undetectable by microscopy and RDT [15, 65]. Efforts towards prompt diagnosis and clinical prevention of malaria (e.g. insecticide-treated net usage, indoor residual spraying, reducing breeding sites, chemotherapy etc.) in these older individuals should be strengthened as they may serve as important reservoirs for continued disease transmission.

The generally high PPV (> 93%) but low NPV (< 76%) from this study indicate that a positive RDT or microscopy test is a good predictor of malaria however, a negative test may not necessarily indicate no infection [66, 67], hence the need for improved diagnosis. Attempts to improve the performance of these point-of-care tests include the development of ultrasensitive rapid diagnostic tests (uRDTs) and regular refresher training to microscopists. uRDTs have been reported to be more sensitive than conventional RDTs although they were not as specific [28, 68, 69]. Alternatively, significant improvement in malaria microscopy has been reported following multiple rounds of refresher training to laboratory professionals [70, 71]. Since microscopy and and RDT remain the best available point-of-care tests in resource limited settings, efforts to sustain and improve their accuracy should be strengthened to facilitate the quick diagnosis and treatment of all malaria cases.

## Conclusion

This study found RDT to be more sensitive and accurate than microscopy for the detection of clinical malaria in Ghana. Nevertheless, both tests missed substantial submicroscopic infections which were detected by *var*ATS qPCR. There is, therefore, a need to adopt measures to improve microscopy and RDT performance via refresher training and development of quality-assured RDTs. The authors recommend further studies to investigate the role of submicroscopic infections in clinical malaria outcomes and also critically assess its contribution to disease transmission in Ghana. As Ghana sets up a cross-parliamentary caucus to accelerate malaria elimination, submicroscopic infections missed at the hospital should be considered an important bottleneck to overcome.

## Supplementary Information

**Additional file 1.** Diagnostic agreement and performance between both experts.

## Data Availability

All data generated or analyzed during the current study are included in this published article [and its supplementary information files]. Additional data may be found at Harvard Dataverse https://doi.org/10.7910/DVN/ZZ2LBN.

## Abbreviations

RDT: Rapid diagnostic test
qPCR: Quantitative polymerase chain reaction
*var*ATS: *var* Gene acidic terminal sequence
HRP-2: Histidine Rich Protein-2
AGH: Agona Government Hospital
MGH: Mankranso Government Hospital
TAB-KNUST: Department of Theoretical and Applied Biology-Kwame Nkrumah University of Science and Technology
ITN: Insecticide-Treated Net
PPV: Positive Predictive Value
NPV: Negative Predictive Value
